# Significantly Enhanced Electromagnetic Interference Shielding Performances of Epoxy Nanocomposites with Long-Range Aligned Lamellar Structures

**DOI:** 10.1007/s40820-022-00949-8

**Published:** 2022-11-15

**Authors:** Lei Wang, Zhonglei Ma, Hua Qiu, Yali Zhang, Ze Yu, Junwei Gu

**Affiliations:** 1 Shaanxi Key Laboratory of Catalysis, School of Chemistry & Environment Science, Shaanxi University of Technology, Hanzhong, 723001 People’s Republic of China; 2 Shaanxi Key Laboratory of Macromolecular Science and Technology, School of Chemistry and Chemical Engineering, Northwestern Polytechnical University, Xi’an, 710072 People’s Republic of China

**Keywords:** Electromagnetic interference shielding, Epoxy nanocomposites, Ti_3_C_2_T_x_, Fe_3_O_4_, Bidirectional aligned three-dimensional conductive networks

## Abstract

High‑efficiency electromagnetic interference (EMI) shielding materials are of great importance for electronic equipment reliability, information security and human health. In this work, bidirectional aligned Ti_3_C_2_T_x_@Fe_3_O_4_/CNF aerogels (BTFCA) were firstly assembled by bidirectional freezing and freeze-drying technique, and the BTFCA/epoxy nanocomposites with long-range aligned lamellar structures were then prepared by vacuum-assisted impregnation of epoxy resins. Benefitting from the successful construction of bidirectional aligned three-dimensional conductive networks and electromagnetic synergistic effect, when the mass fraction of Ti_3_C_2_T_x_ and Fe_3_O_4_ are 2.96 and 1.48 wt%, BTFCA/epoxy nanocomposites show outstanding EMI shielding effectiveness of 79 dB, about 10 times of that of blended Ti_3_C_2_T_x_@Fe_3_O_4_/epoxy (8 dB) nanocomposites with the same loadings of Ti_3_C_2_T_x_ and Fe_3_O_4_. Meantime, the corresponding BTFCA/epoxy nanocomposites also present excellent thermal stability (*T*_heat-resistance index_ of 198.7 °C) and mechanical properties (storage modulus of 9902.1 MPa, Young's modulus of 4.51 GPa and hardness of 0.34 GPa). Our fabricated BTFCA/epoxy nanocomposites would greatly expand the applications of MXene and epoxy resins in the fields of information security, aerospace and weapon manufacturing, etc.

**Supplementary Information:**

The online version contains supplementary material available at 10.1007/s40820-022-00949-8.

## Highlights

- Ti_3_C_2_T_x_@Fe_3_O_4_/CNF aerogels (BTFCA)/epoxy electromagnetic interference (EMI) shielding nanocomposites with long-range aligned lamellar structures were prepared by bidirectional freezing, freeze-drying and vacuum-assisted impregnation of epoxy resins.
- Successful construction of 3D long-range aligned lamellar structures and electromagnetic synergistic effect could significantly increase the EMI shielding effectiveness and reduce the secondary contamination.
- BTFCA/epoxy EMI shielding nanocomposites possessed outstanding EMI shielding effectiveness of 79 dB, and also presented excellent thermal stabilities and mechanical properties.

**Supplementary Information:**

The online version contains supplementary material available at 10.1007/s40820-022-00949-8.

## Graphical abstract

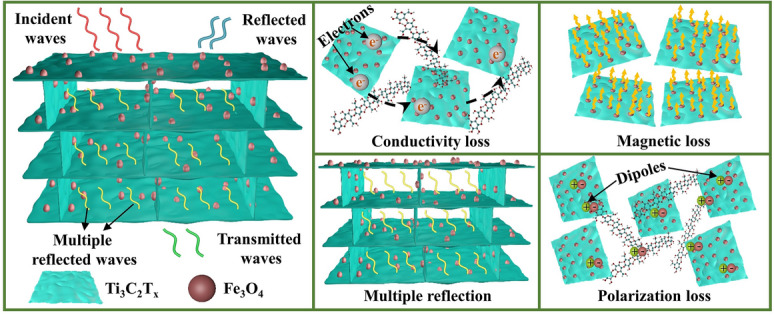

## Introduction

With the rapid development of modern electronic information technology, especially for aerospace weapons and equipment technology, electromagnetic interference (EMI) pollution problem caused by high-frequency and high-power electronic equipment is becoming increasingly serious. It poses serious threats to normal operation of precise electronic components and human health [1–3]. Polymer matrix EMI shielding composites have gradually become the most promising EMI shielding materials due to their advantages of lightweight, excellent specific strength, low cost, easy processing and adjustable performances [4–6].

To our knowledge, polymer matrix EMI shielding composites have achieved satisfactory EMI shielding performances by adding highly conductive and/or magnetic fillers [7–9]. The commonly used conductive fillers are metal, conductive polymers and inorganic nonmetallic materials [10, 11]. Among them, inorganic nonmetallic materials such as the graphite [12], carbon nanotubes (CNT) [13, 14], graphene [15–17] and MXene [18–20] are currently the focus of most attention, due to their advantages of high specific strength, low density, superior electrical conductivity (*σ*) and easy processing, etc. Herein, Ti_3_C_2_T_x_ has been widely applied in the field of EMI shielding due to mature preparation technology and superior *σ* value [21–23]. However, Ti_3_C_2_T_x_ nanosheets tend to agglomerate inner polymer matrix, and have large contact resistance between the nanosheets, leading to higher percolating threshold of the composites [24–26], which would cause machining difficulty and poor mechanical properties [27, 28].

Researches show that construction of three-dimensional (3D) conductive networks is proved to be an effective way to synchronously realize the excellent *σ*, EMI shielding effectiveness (EMI SE) and mechanical properties of polymer composites at relatively low Ti_3_C_2_T_x_ loadings [29–31]. Shi et al. [32] prepared Ti_3_C_2_T_x_ aerogel by freeze-drying method, and further impregnated epoxy resins to prepare Ti_3_C_2_T_x_ aerogel/epoxy composites. When the volume fraction of Ti_3_C_2_T_x_ was 0.40 vol%, the EMI SE of Ti_3_C_2_T_x_ aerogel/epoxy composites was 35 dB. Sun et al. [33] prepared PS@Ti_3_C_2_T_x_ composites by electrostatic self-assembly and molding method. When the mass fraction of Ti_3_C_2_T_x_ was 4.0 wt%, *σ* and EMI SE of PS@Ti_3_C_2_T_x_ composites were 1081 S m^−1^ and 54 dB, respectively. In our previous work, Gu et al. [34] obtained cellulose-derived carbon aerogel@reduced graphene oxide aerogels (CCA@rGO) by freeze-drying and thermal reduction, and further prepared CCA@rGO/polydimethylsiloxane (PDMS) composites by vacuum-assisted impregnation of PDMS. When the mass fraction of CCA@rGO was 3.05 wt%, the *σ* and EMI SE of the obtained CCA@rGO/PDMS composites reached 75 Sm^−1^ and 51 dB, respectively.

Compared with the randomly dispersed 3D conductive networks, the aligned 3D conductive networks are not only conducive to further improving the *σ* [35–37], but also can make efficient utilization of the conductive fillers/polymer interfaces to enhance the reflection and reabsorption of electromagnetic waves [38–40]. Wu et al. [41] prepared Ti_3_C_2_T_x_ foams by directional freezing and further impregnated PDMS to prepare Ti_3_C_2_T_x_ foam/PDMS composites. When the mass fraction of Ti_3_C_2_T_x_ was 6.1 wt%, the *σ* and EMI SE of Ti_3_C_2_T_x_ foam/PDMS composites were 2211 S m^−1^ and 54 dB, respectively. Zhao et al. [15] prepared Ti_3_C_2_T_x_/graphene hybrid aerogels (MGA) by directional freezing, and further impregnated epoxy resins to prepare MGA/epoxy composites. When the volume fraction of graphene and Ti_3_C_2_T_x_ was 0.18 and 0.74 vol%, the *σ* and EMI SE of MGA/epoxy composites were up to 695.9 S m−1 and 50 dB, respectively. Compared with the directional aligned 3D conductive networks, the bidirectional aligned 3D conductive networks can further reduce the percolating threshold and enhance the attenuation of electromagnetic waves by taking advantage of more regular internal interfaces [42–44]. Han et al. [45] prepared Ti_3_C_2_T_x_ foams by bidirectional freezing method. The density of Ti_3_C_2_T_x_ foams was only 11.0 mg cm^−3^, EMI SE and SE/density (SSE) reached 71 dB and 8818 dB cm^3^ g^−1^ respectively, which exceeded the shielding performances of most reported foams. Sambyal et al. [46] prepared Ti_3_C_2_T_x_/CNT foams by bidirectional freezing method. Results showed that the EMI SE of Ti_3_C_2_T_x_/CNT foams (with density of only 2.5 mg cm^−3^) was up to 78 dB. Bidirectional aligned 3D Ti_3_C_2_T_x_ foam and Ti_3_C_2_T_x_/CNT foam have been reported to exhibit excellent EMI shielding performances, but relatively poor mechanical properties have limited their broader applications. Cellulose nanofibers (CNF) possess excellent mechanical properties [47, 48], and could be employed to construct bidirectional aligned 3D conductive networks with Ti_3_C_2_T_x_ via bidirectional freezing method by hydrogen bonds, which would be favor of enhancing the mechanical properties of Ti_3_C_2_T_x_ foams [49–51].

In addition, for common polymer matrix EMI shielding composites, most electromagnetic waves are reflected at the interfaces between composites and air due to impedance mismatch, which would cause electromagnetic pollution to the service environment [52–54]. Researches show that the introduction of magnetic materials would improve the impedance matching of composites and air, weaken the reflection of electromagnetic waves, and absorb electromagnetic waves through magnetic loss [55–57]. Among commonly magnetic fillers, Fe_3_O_4_ shows great application potential in EMI shielding materials due to excellent magnetism and low cost [58–60].

In this work, Ti_3_C_2_T_x_ and Fe_3_O_4_ were firstly assembled by electrostatic interaction, followed by combined with CNF through hydrogen bonding, and bidirectional aligned Ti_3_C_2_T_x_@Fe_3_O_4_/CNF aerogels (BTFCA) were then prepared by bidirectional freezing and freeze-drying technique. Finally, the BTFCA/epoxy nanocomposites were prepared by vacuum-assisted impregnation of epoxy resins. Structures and morphologies of BTFCA were characterized by X-ray diffraction (XRD), Raman spectroscopy, X-ray photoelectron spectroscopy (XPS), transmission electron microscopy (TEM) and scanning electron microscopy (SEM). Furthermore, the effects of Fe_3_O_4_ loadings on *σ*, EMI SE, thermal stabilities and mechanical properties of the BTFCA/epoxy nanocomposites were analyzed in detail, and the corresponding EMI shielding mechanism was investigated.

## Experimental Section

### Fabrication of BTFCA

Fe_3_O_4_ was positively modified by CTAB, followed by mixing with Ti_3_C_2_T_x_ dispersion and freeze-dried (−30 °C, < 2 Pa) for 36 h to get Ti_3_C_2_T_x_@Fe_3_O_4_ hybrid. Different contents of Ti_3_C_2_T_x_@Fe_3_O_4_ were dispersed in 10 mL of 2.5 mg mL^−1^ CNF in a glass vessel by a probe sonication for 10 min in an ice bath, followed by vigorous stirring for 3 h. Then the dispersion was poured in a square mold (side length of 3 cm, height of 5 cm, PDMS wedge with a slope angle of around 15° at the bottom, copper as bottom, nylon as wall), and liquid nitrogen (− 196 °C) was used to freeze the bottom of the cylindrical mold through the intermediary of copper blocks. BTFCA was obtained by freeze-drying at −60 °C with pressure less than 5 Pa, followed by annealed at 400 °C for 2 h at a heating rate of 5 °C s^−1^ in an Ar + 5% H_2_ ambient. Ti_3_C_2_T_x_ content was fixed as 400 mg, the weight ratio of Ti_3_C_2_T_x_/Fe_3_O_4_ was 4/1, 2/1, 1/1, and 1/2, respectively, and the corresponding samples were marked as BTFCA-1, BTFCA-2, BTFCA-3 and BTFCA-4. For comparison, the bidirectional Ti_3_C_2_T_x_/CNF aerogels (BTCA) were also prepared.

### Fabrication of BTFCA/epoxy Nanocomposites

**Fig. 1:**
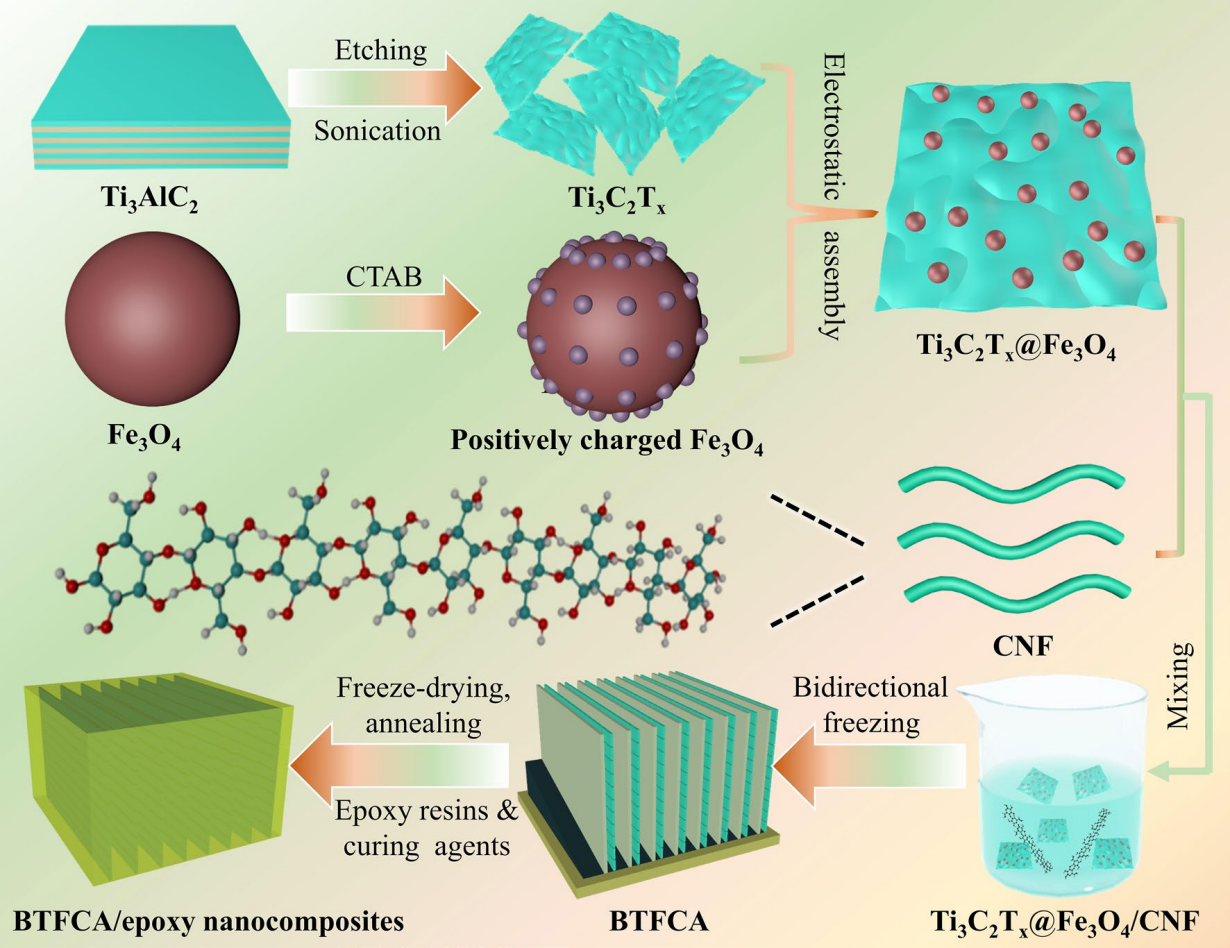
Schematic illustration of fabrication process for BTFCA/epoxy nanocomposites

Epon 862 and diethyl methyl benzene diamine were firstly stirred at 70 °C for 1 h, and then filled into BTFCA via vacuum-assisted impregnation technique. Finally, BTFCA/epoxy nanocomposites were prepared by heating at 120 °C for 5 h. For comparison, BTCA/epoxy nanocomposites were prepared by the same process. Figure 1 is the schematic diagram of preparation for BTFCA/epoxy nanocomposites.

## Results and Discussion

### Characterization of BTCA and BTFCA

**Fig. 2:**
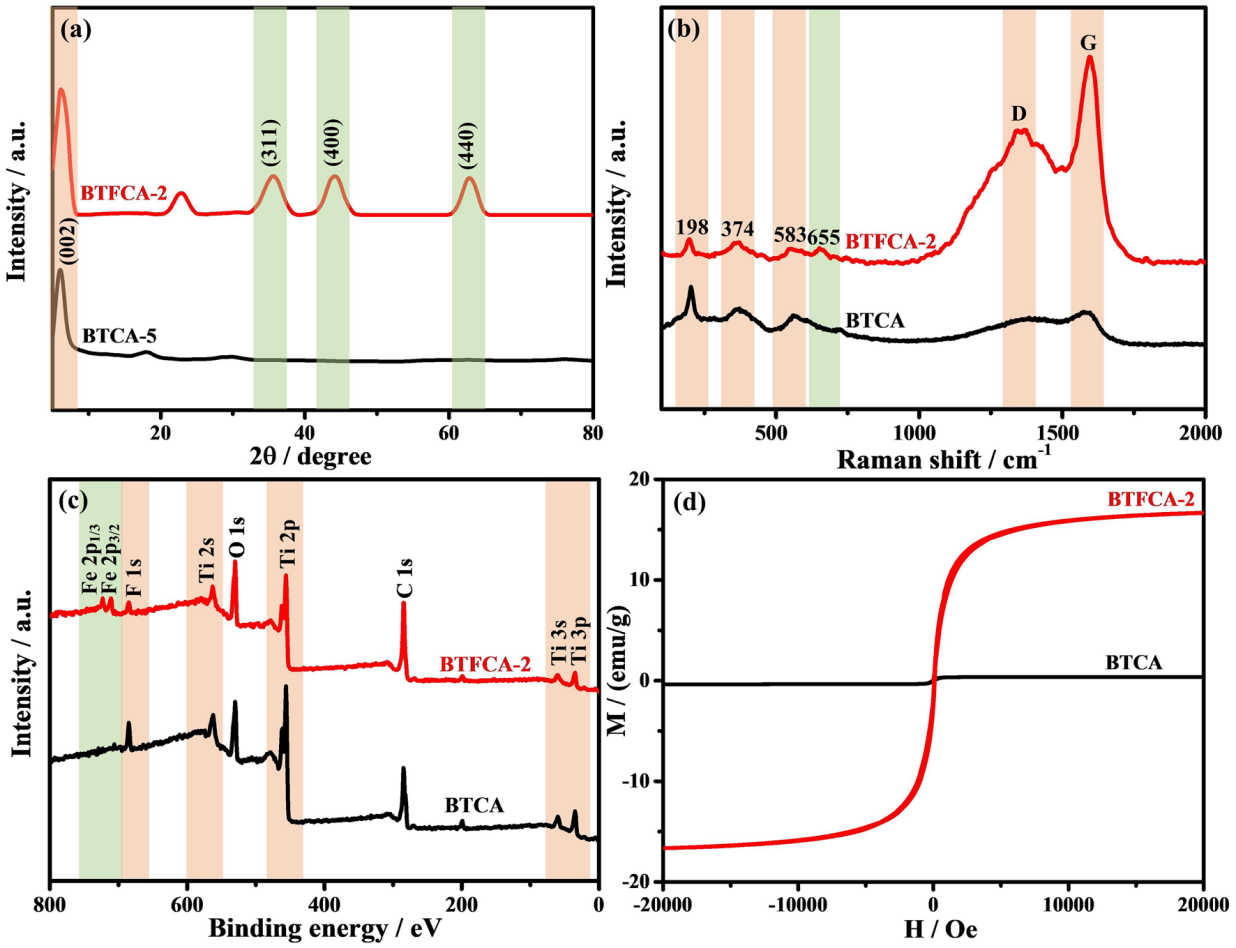
**a** XRD, **b** Raman, **c** XPS spectra and **d** hysteresis loops of BTCA and BTFCA-2

In Fig. S1, Ti_3_AlC_2_ precursor with dense layered structure (Fig. S1a) is exfoliated into few-layered Ti_3_C_2_T_x_ nanosheets (Fig. S1b). In Fig. S2, Fe_3_O_4_ presents (220), (311), (400), (422), (511), and (440) diffraction peaks [61], and corresponding saturation magnetization is 70 emu g^−1^. After electrostatic assembly, Fe_3_O_4_ is uniformly dispersed on Ti_3_C_2_T_x_ nanosheets to obtain Ti_3_C_2_T_x_@Fe_3_O_4_ (Fig. S3). Figure 2 shows XRD, Raman, XPS spectra and hysteresis loops of BTCA and BTFCA-2. From Fig. 2a, BTCA shows diffraction peaks at 6.2° and 23° corresponding to (002) lattice plane of Ti_3_C_2_T_x_ and (002) lattice plane of CNF [62]. After Fe_3_O_4_ is introduced, three new diffraction peaks appear at 36.7°, 43.6°, and 63.8° of BTFCA-2, corresponding to the (311), (400) and (440) crystal planes of Fe_3_O_4_, respectively. As observed in Fig. 2b, BTCA has D-band and G-band at 1355 and 1583 cm^−1^, which are attributed to the graphitized structures [63, 64] formed after thermal reduction of CNF, and characteristic peaks of Ti_3_C_2_T_x_ appear within 100 ~ 700 cm^−1^. Peak at 198 cm^−1^ is attributed to A1g symmetry out-of-plane vibration of Ti atom. Peaks at 374 and 583 cm^−1^ are ascribed to the *E*g group vibration and in-plane (shear) modes of Ti, C, and surfapresentsce functional groups [65]. After Fe_3_O_4_ is introduced, a new peak appears in BTFCA-2 at 655 cm^−1^, which is attributed to the vibration of Fe_3_O_4_ in A1g mode. As shown in Fig. 2c, BTCA presents peaks of Ti 3*p*, Ti 3*s*, C 1*s*, Ti 2*p*, O 1*s*, Ti 2*s*, and F 1*s* at 35, 60, 287, 457, 531, 563, and 685 eV [66]. After Fe_3_O_4_ is introduced, BTFCA-2 shows two new peaks at 709 and 723 eV, which are attributed to Fe 2*p*_3/2_ and Fe 2*p*_1/2_, respectively. From Fig. 2d, the saturation magnetization of BTCA is 0, and the saturation magnetization of BTFCA-2 significantly improves to 16.7 emu g^−1^. XRD, Raman spectroscopy, XPS and hysteresis loops indicate that BTFCA has been successfully prepared.

### Morphologies of BTFCA and BTFCA/epoxy Nanocomposites

**Fig. 3:**
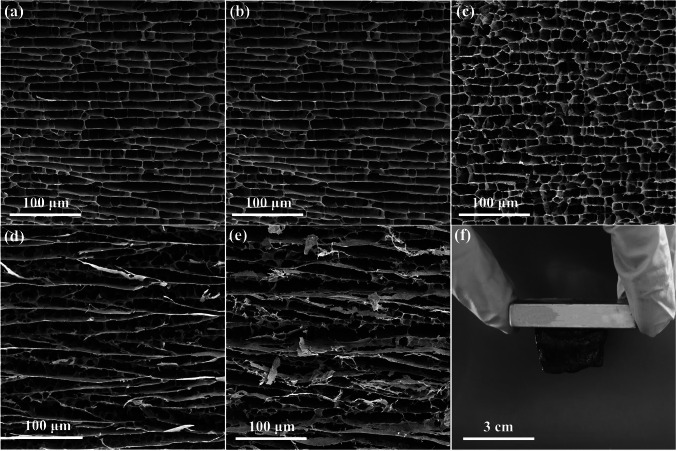
**a** SEM images of BTCA, **b** BTFCA-1, **c** BTFCA-2, **d** BTFCA-3 and **e** BTFCA-4. **f** Digital photograph of magnet attracting BTFCA-2

Figure 3 is the SEM images of BTCA and BTFCA, as well as the photo of BTFCA-2 attracted on the magnet. From Fig. 3a, Ti_3_C_2_T_x_ and CNF in BTCA support each other, to form the long-range aligned lamellar structures. After Fe_3_O_4_ is introduced, BTFCA still maintains the long-range aligned lamellar structures. With low loadings of Fe_3_O_4_, the cell size of BTFCA increases slightly (Fig. 3b-c). However, with the excessive loadings of Fe_3_O_4_, the cell size of BTFCA increases significantly (Fig. 3d-e). The reason is that, in bidirectional freezing process, the temperature difference makes ice crystals grow orderly in both radial and axial directions at the same time. Ti_3_C_2_T_x_, Fe_3_O_4_ and CNF are forced to align along the direction of ice crystal growth. After removal of ice crystals by freeze-drying, the long-range aligned lamellar structures are formed in BTFCA. The addition of Fe_3_O_4_ would destroy the hydrogen bonds between Ti_3_C_2_T_x_ and CNF to some extent. With low loadings of Fe_3_O_4_, Ti_3_C_2_T_x_ and CNF still form abundant hydrogen bonding to maintain the long-range aligned lamellar structures, only causing slight increase in cell size of BTFCA. The addition of excessive Fe_3_O_4_ would damage the hydrogen bonds between Ti_3_C_2_T_x_ and CNF, which reduces the overlap between Ti_3_C_2_T_x_ and CNF, resulting in great increase in cell size of BTFCA. In addition, BTFCA-2 can overcome its own gravity by magnetic force and attract to the magnet (Fig. 3f), indicating that the introduction of Fe_3_O_4_ endows BTFCA with outstanding magnetism. Figure S4 shows the SEM images of BTCA/epoxy and BTFCA-2/epoxy nanocomposites. Both BTCA/epoxy and BTFCA-2/epoxy nanocomposites can well maintain the original long-range aligned lamellar structures, indicating that the mechanical properties are strong enough to resist the adhesion force generated by impregnation of epoxy resins and maintain the structural integrity. On the one hand, Ti_3_C_2_T_x_ nanosheets and CNF possess numbers of polar functional groups such as -OH and -F on the surface to form abundant hydrogen bonds, which is conducive to enhancing the stiffness of BTFCA. On the other hand, the high rigidity of Ti_3_C_2_T_x_ nanosheets and Fe_3_O_4_ also endows BTFCA with great rigidity, which enables BTFCA/epoxy nanocomposites to maintain the integrity of the long-range aligned lamellar structures.

### σ and EMI Shielding Performances of BTFCA/epoxy Nanocomposites

**Fig. 4:**
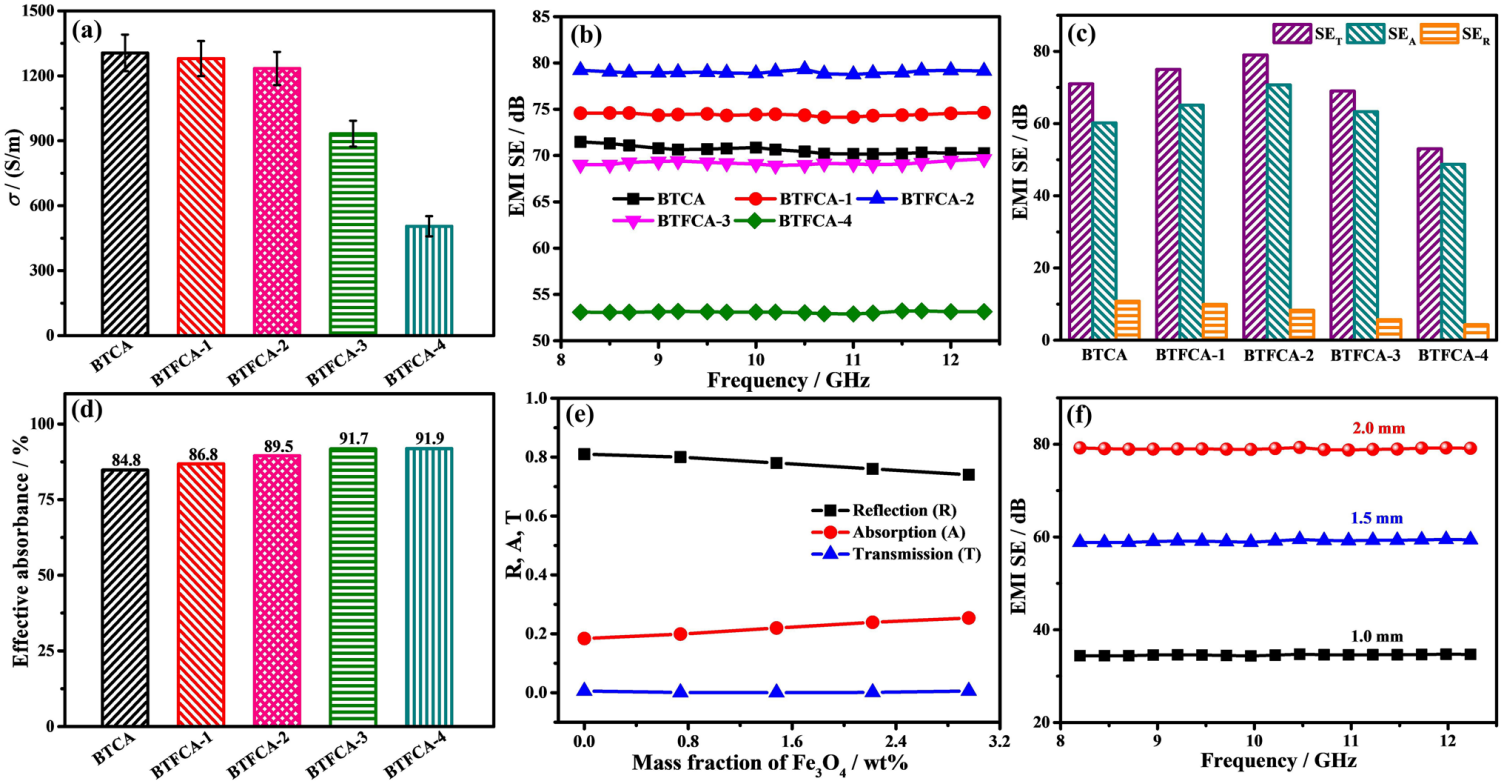
**a** σ, **b** EMI SE, **c** corresponding *SE*_T_, *SE*_A_, *SE*_R_, **d** effective absorbance and **e** R, A, T coefficients of BTCA/epoxy and BTFCA/epoxy nanocomposites. **f** EMI SE of BTFCA-2/epoxy nanocomposites in different thicknesses

Figure 4a shows the *σ* of BTCA/epoxy and BTFCA/epoxy nanocomposites, and the relevant values are shown in Tab S1. The *σ* of BTFCA/epoxy nanocomposites decreases gradually with increasing loadings of Fe_3_O_4_. When the mass fraction of Fe_3_O_4_ is 1.48 wt%, the *σ* of BTFCA-2/epoxy nanocomposites is 1235 S m^−1^, lower than that of BTCA/epoxy (1306 S m^−1^) nanocomposites, and also significantly higher than that of blended Ti_3_C_2_T_x_@Fe_3_O_4_/epoxy (7.6 S m^−1^, Tab. S1) nanocomposites with the same loadings of Ti_3_C_2_T_x_ and Fe_3_O_4_. Highly conductive Ti_3_C_2_T_x_ nanosheets are aligned along the axial and radial directions in BTCA and BTFCA to construct a bidirectional aligned 3D conductive network, which enhances the contact among Ti_3_C_2_T_x_ nanosheets and forms abundant conductive paths, thus showing outstanding *σ*. The introduction of Fe_3_O_4_ would affect the contact among Ti_3_C_2_T_x_ nanosheets and hinder formation of Ti_3_C_2_T_x_-Ti_3_C_2_T_x_ conductive paths, leading to slightly reduced *σ* of BTFCA/epoxy nanocomposites. The addition of excessive Fe_3_O_4_ reduces the overlap among Ti_3_C_2_T_x_ nanosheets and significantly increases the cell size of BTFCA, which severely restricts the formation of bidirectional aligned 3D conductive networks for BTFCA, resulting in significant decrease of *σ*.

**Fig. 5:**
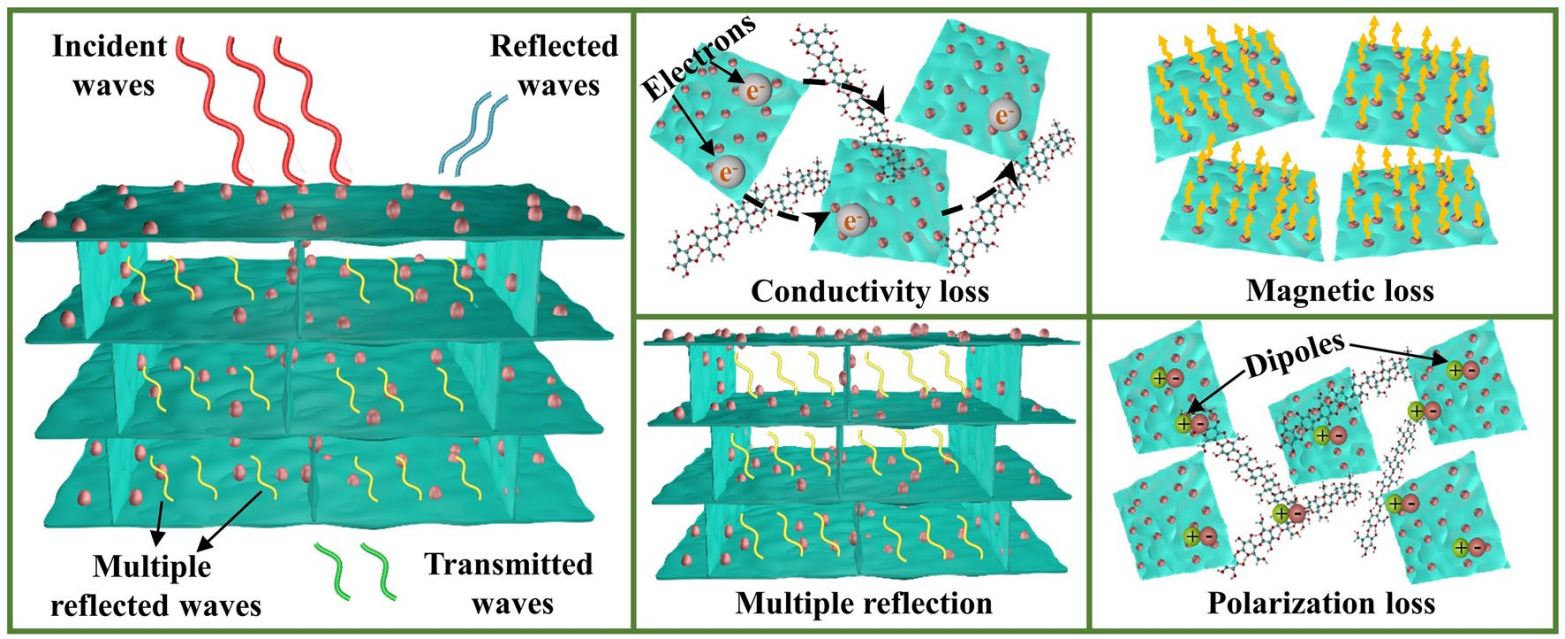
Schematic illustration of EMI shielding mechanism for BTFCA/epoxy nanocomposites

Figure 4b illustrates EMI SE of BTCA/epoxy and BTFCA/epoxy nanocomposites. The EMI SE of BTFCA/epoxy nanocomposites increases first and then decreases with increasing loadings of Fe_3_O_4_. When the mass fraction of Fe_3_O_4_ is 1.48 wt%, EMI SE of BTFCA-2/epoxy nanocomposites is 79 dB, 11.3% higher than that of BTCA/epoxy (71 dB) nanocomposites, and also about 10 times that of blended Ti_3_C_2_T_x_@Fe_3_O_4_/epoxy (8 dB, Fig. S5) nanocomposites with the same loadings of Ti_3_C_2_T_x_ and Fe_3_O_4_. The bidirectional aligned 3D conductive networks of BTFCA/epoxy nanocomposites provide abundant moving loads such as charge carriers. Under alternating electric field, they can induce microcurrent to form electrical loss through tunneling effect and other ways, and convert the energy of electromagnetic waves into heat. At the same time, the internal complex heterogeneous interfaces in BTFCA/epoxy nanocomposites extend the transmission paths of electromagnetic waves, which are conducive to enhancing the scattering and reabsorption of electromagnetic waves and further dissipating electromagnetic waves. After introduction of Fe_3_O_4_, reduced overlaps among Ti_3_C_2_T_x_ nanosheets lead to gradually decreased *σ* of BTFCA/epoxy nanocomposites and weakened electrical loss (such as ohmic loss) is not conducive to improvement of EMI SE. On the other hand, BTFCA/epoxy nanocomposites construct 3D magnetic networks, which enhances the multiple reflection and reabsorption of electromagnetic waves and strengthens the magnetic hysteresis loss and other magnetic losses of electromagnetic waves, so as to improve the dissipation ability of electromagnetic waves. In addition, the introduction of Fe_3_O_4_ brings more heterogeneous interfaces. Due to interface polarization, there are a large number of dipoles at heterogeneous interfaces, which will cause polarization loss to electromagnetic waves and further enhance the attenuation of electromagnetic waves. As a result, BTFCA-2/epoxy nanocomposites present the best EMI shielding performances. And the corresponding schematic illustration of EMI shielding mechanism of BTFCA/epoxy nanocomposites is shown in Fig. 5.

Figure 4c is *SE*_T_, *SE*_A_ and *SE*_R_ of BTCA/epoxy and BTFCA/epoxy nanocomposites. With increasing loadings of Fe_3_O_4_, *SE*_R_ of BTFCA/epoxy nanocomposites decreases gradually, and *SE*_A_ increases first and then decreases. When the mass fraction of Fe_3_O_4_ is 1.48 wt%, *SE*_R_ and *SE*_A_ of BTFCA-2/epoxy nanocomposites are 8 and 71 dB respectively. With increasing loadings of Fe_3_O_4_, the gradually decreased *σ* of BTFCA/epoxy nanocomposites improves the impedance matching, resulting in gradual decrease of *SE*_R_. Although the electrical loss of BTFCA/epoxy nanocomposites to electromagnetic waves is gradually weakened, the internal multiple reflection, magnetic loss and polarization loss are gradually enhanced. As a result, *SE*_A_ of BTFCA-2/epoxy nanocomposites is the maximum.

Figure 4d shows the electromagnetic wave effective absorbance of BTCA/epoxy and BTFCA/epoxy nanocomposites. With increasing loadings of Fe_3_O_4_, the electromagnetic wave effective absorbance of BTFCA/epoxy nanocomposites gradually increases. When the mass fraction of Fe_3_O_4_ is 1.48 wt%, the electromagnetic wave effective absorbance of BTFCA-2/epoxy nanocomposites is 89.5%. The introduction of Fe_3_O_4_ not only improves the impedance matching of BTFCA/epoxy nanocomposites and air, but also enhances the magnetic loss to improve the electromagnetic wave effective absorbance.

Figure 4e is the reflection (*R*), absorption (*A*) and transmission (*T*) coefficients of BTCA/epoxy and BTFCA/epoxy nanocomposites. With increasing loadings of Fe_3_O_4_, the *R* coefficient of BTFCA/epoxy nanocomposites decreases gradually, and the *T* coefficient decreases first and then increases. When the mass fraction of Fe_3_O_4_ is 1.48 wt%, the *T* coefficient of BTFCA-2/epoxy nanocomposites is the lowest, only 4 × 10^–4^, and the *R* coefficient is 0.78. It demonstrates that the incorporation of Fe_3_O_4_ can significantly improve the EMI shielding performances. With increasing loadings of Fe_3_O_4_, the *σ* of BTFCA/epoxy nanocomposites decreases gradually, which improves the impedance matching between BTFCA/epoxy nanocomposites and the air, and reduces the *R* coefficient gradually, thus weakening the secondary electromagnetic pollution. With introduction of Fe_3_O_4_, although the decreased *σ* leads to reduced electrical loss to electromagnetic waves, the internal multiple reflection, magnetic loss and interfacial polarization loss are enhanced. Under the comprehensive action, BTFCA-2/epoxy nanocomposites show the optimal EMI shielding performances and the corresponding *T* coefficient is the lowest.

Figure 4f demonstrates EMI SE of BTFCA-2/epoxy nanocomposites in different thicknesses, and the EMI SE of BTFCA/epoxy nanocomposites increases with increasing thicknesses. When the thickness increases from 1 to 2 mm, the EMI SE of BTFCA-2/epoxy nanocomposites increases from 34 to 79 dB. This is because the increased thickness is in favor of lengthening the propagation path of electromagnetic waves in BTFCA/epoxy nanocomposites, which is conducive to the scattering and reabsorption of electromagnetic waves to further enhance EMI shielding performances.

### Thermal Stabilities of BTFCA/epoxy Nanocomposites

**Fig. 6:**
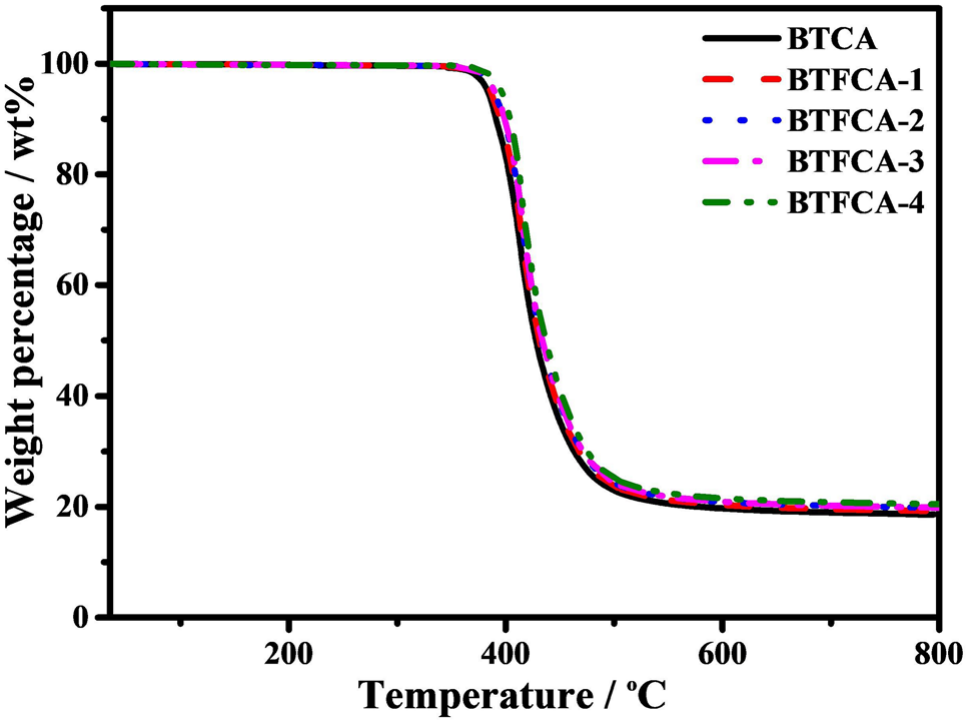
TGA curves of BTCA/epoxy and BTFCA/epoxy nanocomposites

Figure 6 presents the thermogravimetric analysis (TGA) curves of BTCA/epoxy and BTFCA/epoxy nanocomposites, and the corresponding thermal data are shown in Tab. S2. *T*_heat-resistance index_ (*T*_HRI_) can reflect the heat resistance of the materials [67]. It can be seen that *T*_HRI_ of BTFCA/epoxy nanocomposites increases gradually with increasing loadings of Fe_3_O_4_. When the mass fraction of Fe_3_O_4_ is 1.48 wt%, *T*_HRI_ of BTFCA-2/epoxy nanocomposites is 198.7 °C, increased by 2.6 °C compared with that of BTCA/epoxy (196.1 °C) nanocomposites. The main reason is that Fe_3_O_4_ has more excellent heat resistance and relatively lower weight loss, resulting in slightly improved thermal stabilities of BTFCA/epoxy nanocomposites.

### Mechanical Properties of BTFCA/epoxy Nanocomposites

**Fig. 7:**
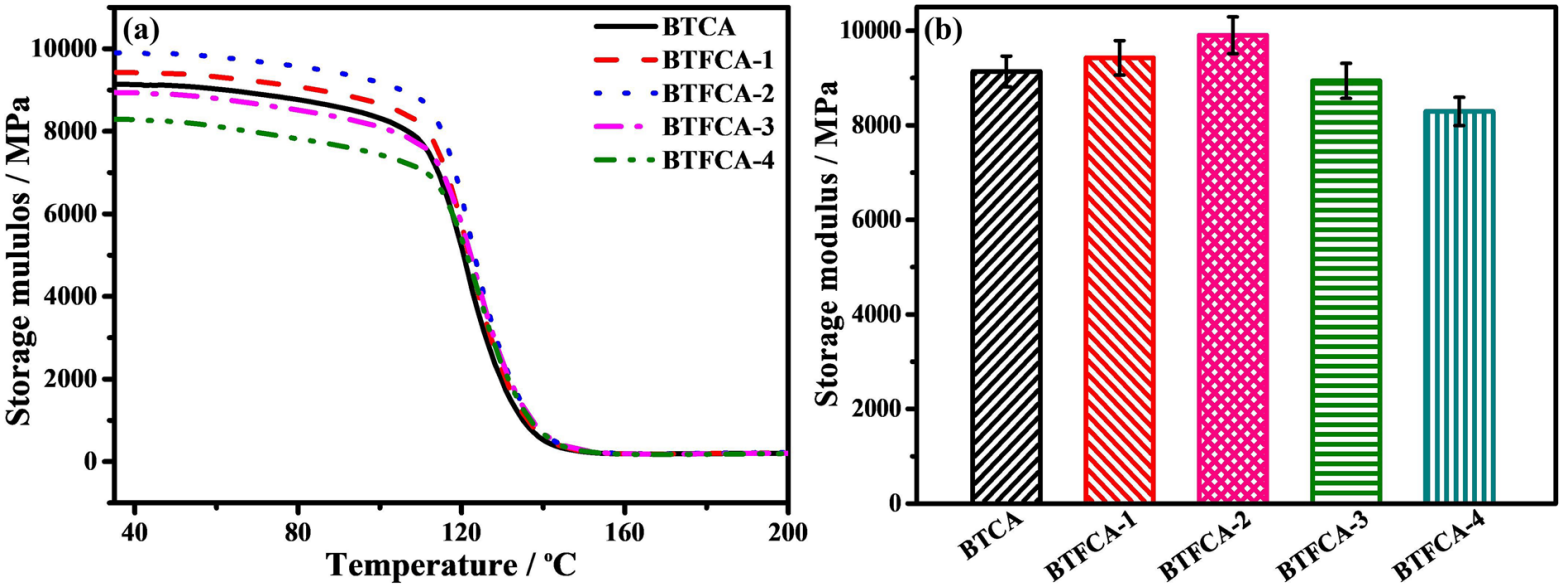
**a** DMA curves and **b** storage modulus of BTCA/epoxy and BTFCA/epoxy nanocomposites

Figures 7 and S6 show storage modulus and Tan *δ* of BTCA/epoxy and BTFCA/epoxy nanocomposites, the corresponding data are given in Tab. S2. The storage modulus of BTFCA/epoxy nanocomposites increases first and then decrease with increasing loadings of Fe_3_O_4_. When the mass fraction of Fe_3_O_4_ is 1.48 wt%, the storage modulus of BTFCA-2/epoxy nanocomposites is 9902.1 MPa, higher than that of BTCA/epoxy (9137.3 MPa) nanocomposites. It is due to that the introduction of Fe_3_O_4_ increases the surface roughness of BTFCA, which is conducive to enhancing interfacial strength between BTFCA and epoxy resins, thus improving the storage modulus. However, the addition of excessive Fe_3_O_4_ would destroy the bidirectional aligned 3D networks of BTFCA, leading to decreased storage modulus.

**Fig. 8:**
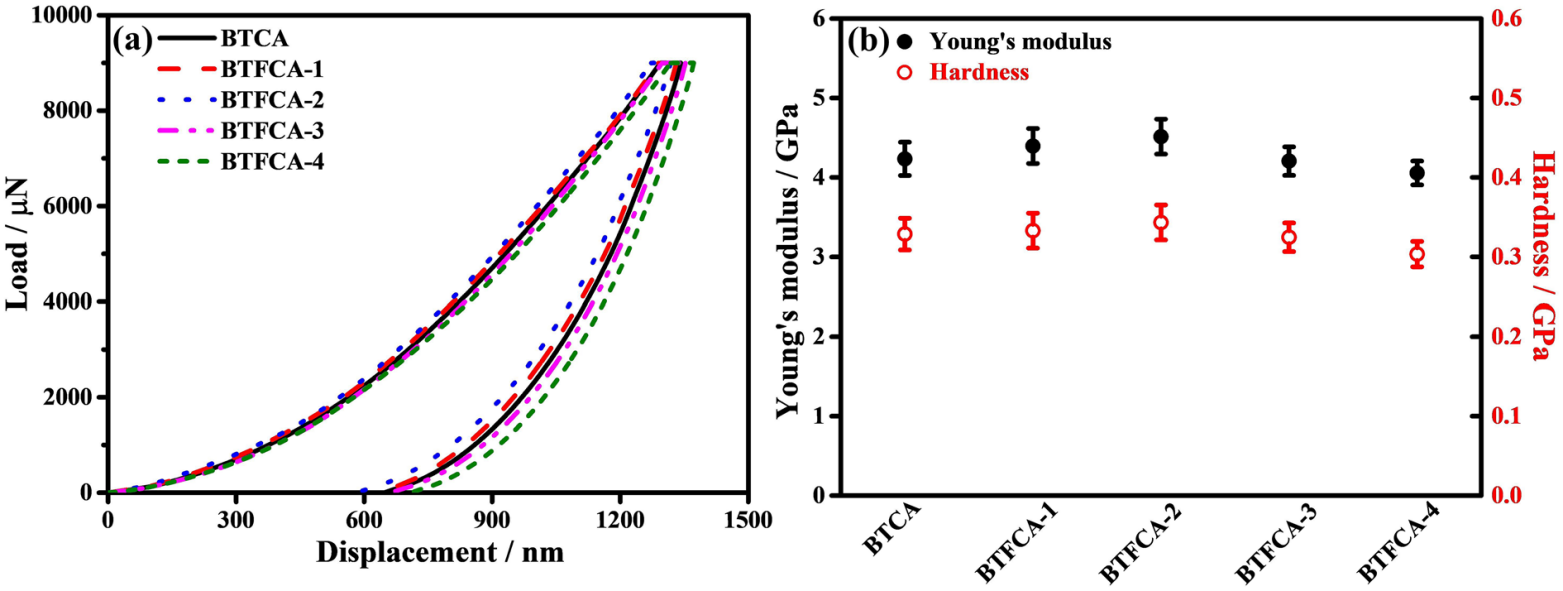
**a** Representative load-displacements curves, **b** Young’s modulus and hardness of BTCA/epoxy and BTFCA/epoxy nanocomposites

Figure 8 presents the load–displacement curves, Young's modulus and hardness of BTCA/epoxy and BTFCA/epoxy nanocomposites, and the corresponding data are given in Tab. S2. Young's modulus and hardness of BTFCA/epoxy nanocomposites increase first and then decrease with increasing loadings of Fe_3_O_4_. When the mass fraction of Fe_3_O_4_ is 1.48 wt%, the Young's modulus and hardness of BTFCA-2/epoxy nanocomposites are 4.51 and 0.34 GPa, improved by 6.9% and 6.3% than those of BTCA/epoxy (4.23 and 0.32 GPa) nanocomposites. The reason is that the high stiffness and hardness of Fe_3_O_4_ can improve the Young's modulus and hardness of BTFCA/epoxy nanocomposites. However, the addition of excessive Fe_3_O_4_ would damage the bidirectional aligned 3D networks of BTFCA and weaken the ability of BTFCA/epoxy nanocomposites to resist deformation under external force, resulting in decreased Young’s modulus and hardness.

## Conclusion

Bidirectional aligned BTFCA and the corresponding BTFCA/epoxy nanocomposites with long-range aligned lamellar structures were successfully prepared. Benefitting from the successful construction of bidirectional aligned 3D conductive networks and electromagnetic synergistic effect, when the mass fraction of Ti_3_C_2_T_x_ and Fe_3_O_4_ is 2.96 and 1.48 wt%, BTFCA/epoxy nanocomposites show outstanding EMI SE of 79 dB, about 10 times of that of blended Ti_3_C_2_T_x_@Fe_3_O_4_/epoxy (8 dB) nanocomposites with the same loadings of Ti_3_C_2_T_x_ and Fe_3_O_4_. Moreover, BTFCA/epoxy nanocomposites also present excellent thermal stability (*T*_HRI_ of 198.7 °C) and mechanical properties (storage modulus of 9902.1 MPa, Young's modulus of 4.51 GPa, and hardness of 0.34 GPa). The obtained BTFCA/epoxy nanocomposites would greatly expand the applications of MXene and epoxy resins in the fields of information security, aerospace and weapon manufacturing, etc.

## Supplementary Information

Below is the link to the electronic supplementary material.

Supplementary file1 (PDF 613 KB)
