# Supplementary material for: Significantly Enhanced Electromagnetic Interference Shielding Performances of Epoxy Nanocomposites with Long-Range Aligned Lamellar Structures
Source: Nanomicro Lett. 2022 Nov 15;14:224. doi: 10.1007/s40820-022-00949-8 (PMC9666581; doi:10.1007/s40820-022-00949-8)
Supplement: Supplementary file 1 — Supplementary file1 (PDF 613 KB) [file 40820_2022_949_MOESM1_ESM.pdf]

Supporting Information for

Significantly Enhanced Electromagnetic Interference Shielding Performances of Epoxy Nanocomposites with Long-Range Aligned Lamellar Structures

Lei Wang[1, 2], Zhonglei Ma[2], Hua Qiu[2, *], Yali Zhang[2], Ze Yu[2], Junwei Gu[2, *]

[1] Shaanxi Key Laboratory of Catalysis, School of Chemistry & Environment Science, Shaanxi University of Technology, Hanzhong 723001, P. R. China

[2] Shaanxi Key Laboratory of Macromolecular Science and Technology, School of Chemistry and Chemical Engineering, Northwestern Polytechnical University, Xi'an 710072, P. R. China

*Corresponding authors. E-mail: huaqiu@nwpu.edu.cn (H. Qiu); gjw@nwpu.edu.cn or nwpugjw@163.com (J. Gu)

S1 Experimental Section

S1.1 Main Materials

Bisphenol F epoxy (Epon 862) was provided by Hexion Inc (Columbus Co., USA). Diethyl methyl benzene diamine was purchased from Baiduchem Co., Ltd. (Hubei, China). Ti_3AlC_2 powder (38 μm, 98% purity) was supplied by 11 Technology Co., Ltd. (Jilin, China). Concentrated HCl and LiF were both bought from Macklin (Shanghai Co., China). $FeCl_3·6H_2O$, $FeCl_2·4H_2O$, NaOH and cetyl trimethyl ammonium bromide (CTAB) were all gotten from J&K chemical (Beijing Co., China). Cellulose nanofibers (CNF, 4~10 nm of diameter and 1~3 μm of length) were received from Qihong Technology Co., Ltd. (Guangxi Co., China).

S1.2 Fabrication of $Ti_3C_2T_x$ Nanosheets

$Ti_3C_2T_x$ was fabricated by modified minimally intensive layer delamination method. Etching solution was prepared by dissolving 1.6 g of LiF in 20 mL of 9 M HCl. Then 1.0 g of Ti_3AlC_2 powder was gradually added into the mixed solution in an ice bath within 5 min, which was then stirred at 500 rpm and kept reaction at 35 °C for 24 h. Subsequently, the obtained product was centrifuged with deionized water at 3500 rpm for 5 min for each cycle until pH was about 7, to observe the dark-green supernatant. The concentrated supernatant of $Ti_3C_2T_x$ nanosheets was then sonicated by a probe sonicator (300 W) for 5 min. Finally, the few-layered $Ti_3C_2T_x$ could be obtained by centrifugation at 3500 rpm for 1 h, followed by freeze-drying treatment of the supernatant solution.

S1.3 Fabrication of Fe_3O_4 Nanoparticles

1.0 g $FeCl_2·4H_2O$ and 2.7 g $FeCl_3·6H_2O$ were dissolved in 12.5 mL 0.8 M HCl, and the mixtures were added drop by drop into 125 mL 1.5 M NaOH solution at 80 °C at nitrogen atmosphere and stirred vigorously until black precipitate was produced. Fe_3O_4 could be obtained by washing the black precipitate with deionized water.

S1.4 Characterizations

X-ray diffraction (XRD) of the samples was tested on a Shimadzu-7000 type X-ray diffraction (Shimadzu, Japan, λ = 0.154 nm). X-ray photoelectron spectroscopy (XPS) analyses of the samples were collected on a PHI5400 equipment (PE Corp., England). Raman spectrum of the samples was measured on a WITec Alpha300R (PE Corp., England) with a He-Ne laser, tuned at 532 nm. Thermogravimetric analyses (TGA) of the samples were carried out using STA 449F3 (Netzsch C Corp., Germany) at 10 ^{o}C min^{-1} at argon atmosphere over the entire temperature range (40～800 ^{o}C). Dynamic mechanical analyses (DMA) of the samples were performed by DMA/SDTA861e (METTLER TOLEDO Corp., Switzerland) with a frequency of 1 Hz and a heating rate of 5 ^{o}C min^{-1} in the temperature range of 35～200°C, and the corresponding specimen dimension was of 50.00 × 10.00 × 3.00 mm^{3}. Scanning electron microscopy (SEM) images of the samples were captured on a VEGA3-LMH equipment (TESCAN Co., Czech Republic). Transmission electron microscope (TEM) images of the samples were obtained on a Talos F200X/TEM microscope (FEI Co., USA) operated at 200 kV. Magnetic properties of the samples were tested by CFMS-14T physical property measurement system (Cryogenic Co., UK). Electrical conductivities (σ) values of the samples were analyzed using RTS-8 (Guangzhou Four Probes Technology Corp., China). EMI shielding performances of the samples were measured by an MS4644A Vector Network Analyzer instrument (Anritsu Corp., Japan), which used the wave-guide method in the X-band frequency range according to ASTMD5568-08, and the corresponding specimen dimension was 22.86 × 10.16 × 2.00 mm^{3}. The electromagnetic wave effective absorbance represents the proportion of absorption loss in the total EMI SE, and can be expressed as:

$$\text{Effective absorbance} = SE_{\mathrm{A}}/SE_{\mathrm{T}} \times 100\% \qquad \text{(S1)}$$

Nanoindentation tests of the samples were obtained by using a Hysitron TI-980 TriboIndenter (Hysitron Co., USA). The peak indentation load was fixed at 9 mN and the unloading rate was 300 mN/s and 450 mN/s, respectively.

S2 Supplementary Figures and Tables

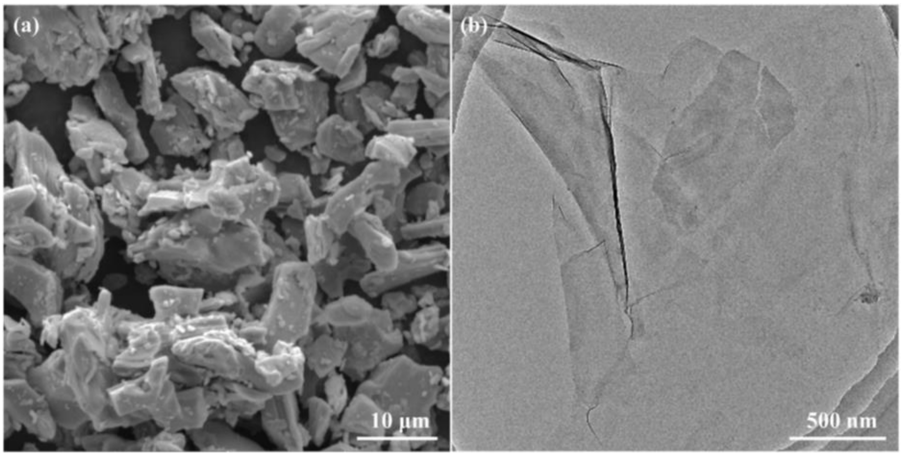

Fig. S1 SEM image of Ti_3AlC_2 (**a**) and TEM image of $Ti_3C_2T_x$ (**b**)

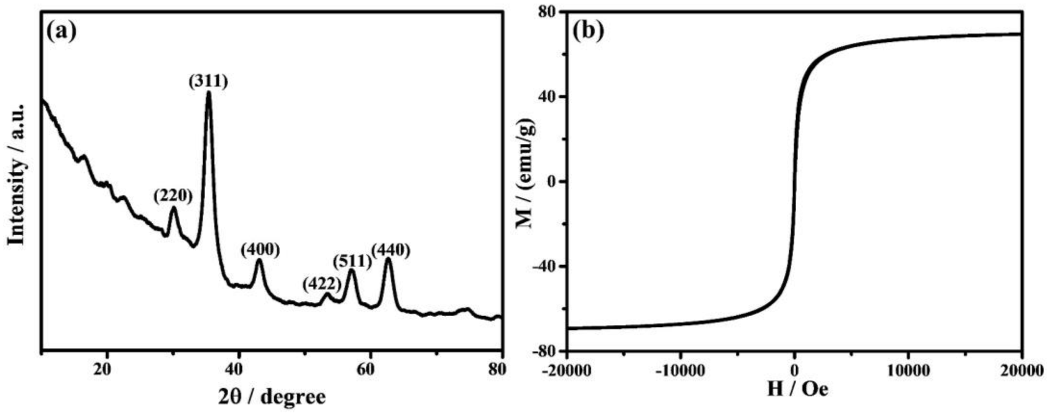

Fig. S2 XRD pattern (**a**) and magnetization curve (**b**) of Fe_3O_4 nanoparticles

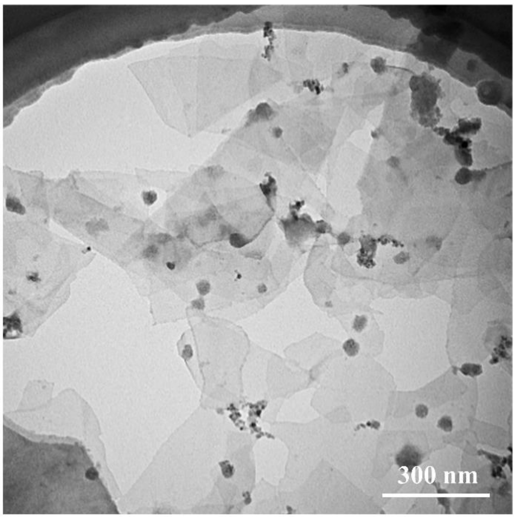

Fig. S3 TEM image of $Ti_3C_2T_x$@Fe_3O_4

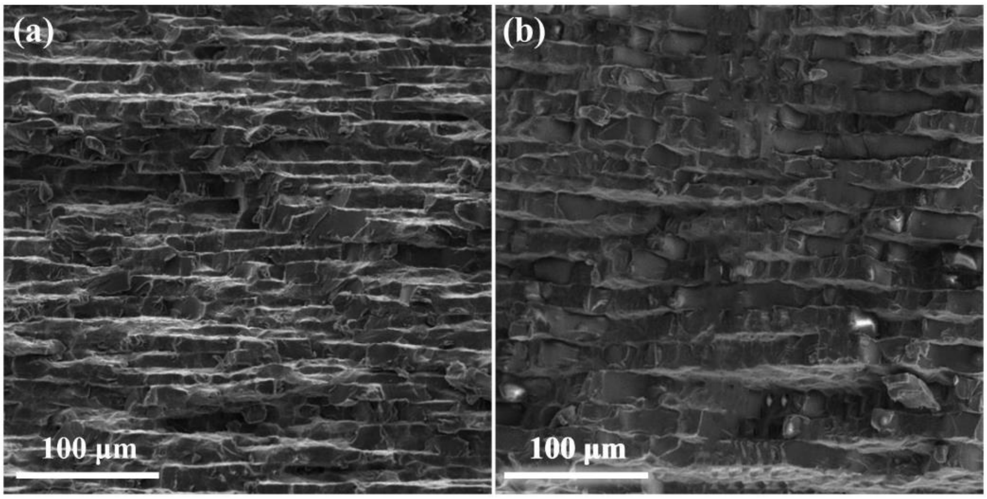

Fig. S4 Morphologies of BTFCA-2/epoxy nanocomposites. SEM images of BTCA/epoxy (**a**) and BTFCA-2/epoxy (**b**) nanocomposites

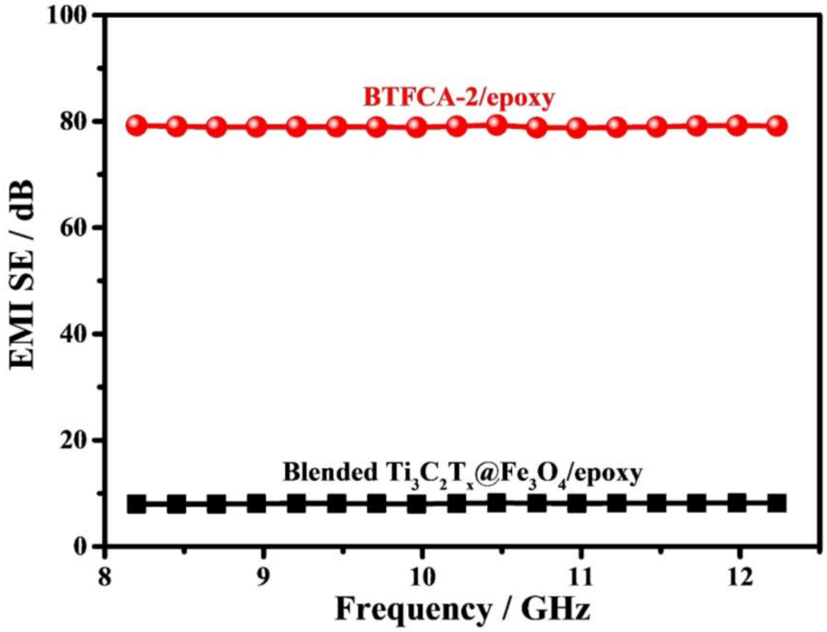

Fig. S5 EMI SE of BTFCA-2/epoxy and blended $Ti_3C_2T_x@Fe_3O_4$/epoxy nanocomposites with the same fillers loading

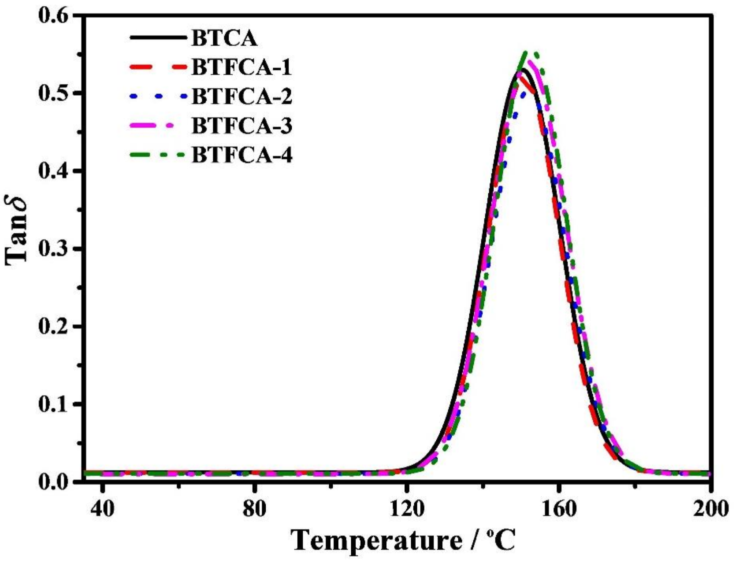

Fig. S6 Tanδ of BTCA/epoxy and BTFCA/epoxy nanocomposites

Table S1 Comparison of σ and EMI SE for BTCA/epoxy and BTFCA/epoxy nanocomposites

Samples	$Ti_3C_2T_x$ content / wt%	Fe_3O_4 content / wt%	σ / (S/m)	EMI SE / dB
Blended $Ti_3C_2T_x@Fe_3O_4$/epoxy	2.96	1.48	7.6	8
BTCA/epoxy	2.96	0	1306	71
BTFCA-1/epoxy	2.96	0.74	1280	74
BTFCA-2/epoxy	2.96	1.48	1235	79
BTFCA-3/epoxy	2.96	2.96	933	69
BTFCA-4/epoxy	2.96	5.92	505	53

Table S2 Thermal characteristic parameters, storage modulus, Young's modulus and hardness of BTCA/epoxy and BTFCA/epoxy nanocomposites

**Samples**	**Weight loss temperature / °C**		**T_{HRI} / °C**	**Storage modulus / MPa**	**Young's modulus / GPa**	**Hardness / GPa**
	**T_5**	**T_{30}**				
BTCA/epoxy	383.6	411.4	196.1	9137.3	4.23	0.32
BTFCA-1/epoxy	386.4	414.5	197.6	9425.4	4.40	0.33
BTFCA-2/epoxy	389.5	416.0	198.7	9902.1	4.51	0.34
BTFCA-3/epoxy	391.8	417.3	199.5	8938.7	4.21	0.32
BTFCA-4/epoxy	393.4	418.7	200.2	8290.9	4.06	0.30

*Sample's heat-resistance index is calculated by Eq. S2.

$$T_{Heat\text{-}resistance\ index}=0.49*[T_5+0.6*(T_{30}-T_5)] \qquad \text{(S2)}$$

T_5 and T_{30} are corresponding decomposition temperature of 5% and 30% weight loss, respectively.
